# Eosinopenia in COVID-19 Patients: A Retrospective Analysis

**DOI:** 10.3390/microorganisms8121929

**Published:** 2020-12-04

**Authors:** Narcisse Ndieugnou Djangang, Lorenzo Peluso, Marta Talamonti, Antonio Izzi, Pierre Alain Gevenois, Alessandra Garufi, Jean-Christophe Goffard, Sophie Henrard, Paolo Severgnini, Jean-Louis Vincent, Jacques Creteur, Fabio Silvio Taccone

**Affiliations:** 1 Department of Intensive Care, Clinique Universitaire de Bruxelles Hôpital Erasme, 1070 Brussels, Belgium; ndieugmou@gmail.com (N.N.D.); marty.talamonti@gmail.com (M.T.); antonio_izzi2010@libero.it (A.I.); garufi.ale@gmail.com (A.G.); jlvincent@intensive.org (J.-L.V.); jacques.creteur@erasme.ulb.ac.be (J.C.); ftaccone@ulb.ac.be (F.S.T.); 2 Department of Radiology, Clinique Universitaire de Bruxelles Hôpital Erasme, 1070 Brussels, Belgium; PierreAlain.Gevenois@erasme.ulb.ac.be; 3 Department of Internal Medicine, Clinique Universitaire de Bruxelles Hôpital Erasme, 1070 Brussels, Belgium; Jean-Christophe.Goffard@erasme.ulb.ac.be (J.-C.G.); Sophie.Henrard@erasme.ulb.ac.be (S.H.); 4 Dipartimento Biotecnologie e Scienze della Vita, Università degli studi dell’ Insubria, U.O. Anestesia e Rianimazione Cardiologica ASST Sette Laghi, 21100 Varese, Italy; paolo.severgnini@uninsubria.it

**Keywords:** coronavirus, eosinophils, chest CT imaging, reverse transcription polymerase, COVID-19

## Abstract

*Objectives*: The aim of this study was to assess the diagnostic role of eosinophils count in COVID-19 patients. *Methods*: Retrospective analysis of patients admitted to our hospital with suspicion of COVID-19. Demographic, clinical and laboratory data were collected on admission. Eosinopenia was defined as eosinophils < 100 cells/mm^3^. The outcomes of this study were the association between eosinophils count on admission and positive real-time reverse transcription polymerase chain reaction (rRT-PCR) test and with suggestive chest computerized tomography (CT) of COVID-19 pneumonia. *Results*: A total of 174 patients was studied. Of those, 54% had positive rRT-PCR for SARS-CoV-2. A chest CT-scan was performed in 145 patients; 71% showed suggestive findings of COVID-19. Eosinophils on admission had a high predictive accuracy for positive rRT-PCR and suggestive chest CT-scan (area under the receiver operating characteristic—ROC curve, 0.84 (95% CIs 0.78–0.90) and 0.84 (95% CIs 0.77–0.91), respectively). Eosinopenia and high LDH were independent predictors of positive rRT-PCR, whereas eosinopenia, high body mass index and hypertension were predictors for suggestive CT-scan findings. *Conclusions*: Eosinopenia on admission could predict positive rRT-PCR test or suggestive chest CT-scan for COVID-19. This laboratory finding could help to identify patients at high-risk of COVID-19 in the setting where gold standard diagnostic methods are not available.

## 1. Introduction

At the end of December 2019, the first case of COVID-19 (Coronavirus disease 2019) caused by a novel human coronavirus, called SARS-CoV-2, was reported in Wuhan, China [1]. This virus is an encapsulated β-coronavirus, which contains a single-stranded RNA as a nucleic material, with a size ranging from 26 to 32 Kbs in length [2]. This virus is most closely related to SARS-CoV, another acute-lung-injury causing coronavirus of zoonotic origin and shares a large proportion of its genome also with the Middle-East Respiratory Syndrome (MERS)-associated virus [3]. COVID-19 showed a high diffusion and became rapidly a pandemic and a clinical challenge for the entire world health-care system [4].

On 15 April 2020 more than two million COVID-19 cases were confirmed in more than 100 countries and over 130,000 deaths declared [5]. The symptoms of the viral infection are heterogeneous, ranging from pauci- or asymptomatic forms to severe pneumonias needing hospitalization and, in some cases, intensive care unit (ICU) admission, often for life support therapies [6,7]. In symptomatic patients, fever, cough, fatigue, shortness of breath and myalgia are the most common symptoms [8].

Due to the high contagious power of the virus, rapid and accurate diagnostic tools are required to rapidly identify potentially infected patients; this would avoid further viral diffusion in the population and help to adequately manage patients with moderate or severe symptoms. The gold standard for COVID-19 diagnosis is based on real-time reverse-transcriptase-polymerase chain reaction (rRT-PCR) test from nasopharyngeal swab specimens or, in severe cases, from broncho-alveolar lavage (BAL) [9]. After some false negative results have been reported using this method in the first days after hospital admission [10], chest computed tomography (CT) scan showed a high sensibility to detect early pulmonary abnormalities suggesting COVID-19 and was considered as the primary diagnostic tool in some epidemic areas [11]; the common CT findings are crazy-paving patterns with bilateral and peripheral distribution [11,12]. 

Nevertheless, rRT-PCR requires laboratory facilities and provides results with a delay ranging from few hours to some days. In addition, chest CT-scan is not easily available in all hospitals worldwide and has additional issues, such as costs and the exposure to ionizing radiations. As such, it could be interesting to evaluate whether commonly available laboratory data, combined with clinical evaluation, could help physicians to identify patients at high-risk for COVID-19 diagnosis, in particular in areas with lack of resources. In a small study, eosinopenia and lymphopenia on admission were reported as useful indicators of COVID-19 in patients with typical symptoms and radiological abnormalities [13]. 

Considering the scarce data on this topic, the aim of this study was to assess the potential role of eosinophils count to predict positive rRT-PCR test or suggestive chest CT-scan findings in patients admitted for suspected COVID-19.

## 2. Materials and Methods

### 2.1. Study Design

This monocentric retrospective cohort study was performed in the COVID departments of the Hôpital Erasme in Brussels, Belgium. The study protocol was approved by the local ethics committee (P2020/246), which waived the need for informed written consent because of its retrospective design and since all interventions were part of the standard patients’ care. None of these patients has been included in previously published studies on this topic.

### 2.2. Study Population

We enrolled patients admitted to our hospital from March 10 (i.e., first admitted patient) to 31 March 2020 with the following inclusion criteria: (a) age of 18 years or older; (b) tested using rRT-PCR assay on the nasopharyngeal swab and/or BAL specimens for suspicion of COVID-19; (c) available laboratory exams on the admission to the hospital. Exclusion criteria were patients already admitted in our institution for other reasons (i.e., intrahospital acquisition of COVID-19) and patients transferred from other hospitals. 

### 2.3. Data Collection 

We collected demographics, comorbidities, patients’ characteristics on admission as well as results of rRT-PCR and chest thin-section CT-scan. Chest CT evaluation was performed by one radiologist (PAG), who reported whether the CT-scan was suggestive or not for COVID-19 (i.e., ground-glass opacities, consolidation or crazy-paving patterns) [11,13]. For clinical assessment on admission, we computed a simplified score based on admission symptoms; one point was attributed to each clinical feature, so that it could range from 0 (i.e., no symptoms) to 5 (i.e., all symptoms were present). We considered the following clinical features: (1) dyspnea; (2) flu-like symptoms (i.e., at least one among fever cough, cold, sore throat, myalgia and fatigue); (3) gastrointestinal symptoms (i.e., at least one among vomiting, diarrhea, dysgeusia and nausea) or anosmia; (4) oxygen saturation <94% breathing room air; (5) respiratory rate > 30 breaths per minute. Results of the blood sample tests on admission, including complete blood count (CBC), coagulation and inflammatory markers (i.e., Fibrinogen; D-Dimers; prothrombin time, PT; C-Reactive Protein, CRP; Procalcitonin, PCT; Lactic Dehydrogenase, LDH; Ferritin) as well as microbiological samples, whenever present, were collected. Eosinopenia was defined as eosinophils <100 cells/mm^3^.

### 2.4. Outcome Assessment

The primary endpoint of this study was to assess the association of eosinophils count on admission and a positive rRT-PCR test. Secondary outcomes included the association of eosinophils count on admission and a suggestive chest CT-scan and the identification of predictors of positive rRT-PCR test and suggestive CT-scan. In addition, we assessed whether eosinopenia was able to predict disease severity on admission, according to WHO criteria (i.e., Mild/Moderate vs. Severe/Critical) [14].

### 2.5. Statistical Analysis 

Descriptive statistics were computed for all study variables. A Kolmogorov–Smirnov test was used, and histograms and normal-quantile plots were examined to verify the normality of distribution of continuous variables. Discrete variables were expressed as count (percentage) and continuous variables as mean ± SD or median (25th–75th percentiles), as appropriate. Demographics and clinical differences between groups (rRT-PCR positive vs. negative test; suggestive vs. nonsuggestive chest CT-scan for COVID-19) were assessed using a chi-square test or Fisher’s exact test for categorical variable, and Student’s *t*-test or Mann–Whitney U-test for continuous variable, as appropriate. The discriminative ability of eosinophils to predict a positive rRT-PCR or a suggestive CT-scan was evaluated using receiver operating characteristic (ROC) curves with the corresponding area under the curve (AUROC). Youden’s index was computed to assess the optimal cut-off of eosinophils for sensitivity and specificity to predict positive rRT-PCR or suspected chest CT-scan findings. Multivariable logistic regression analysis with positive rRT-PCR as the dependent variable was performed in all patients. Collinearity between variables (i.e., an index of collinearity higher than 0.3) was excluded prior to modelling; only variables associated with positive rRT-PCR in the univariate analysis (*p* < 0.1) were included in the multivariate model. Odds ratios (OR) with 95% confidence intervals (CI) were computed. A similar approach was used to perform the multivariate analysis with suspected CT-scan as the dependent variable. A *p* < 0.05 was considered as statistically significant. Statistical analyses were performed using the SPSS 25.0 for Macintosh (IBM, Armonk, NY, USA).

### 2.6. Availability of Data and Materials

The datasets used and/or analysed during the current study are available from the corresponding author on reasonable request.

## 3. Results

### 3.1. Study Population

**Figure 1:**
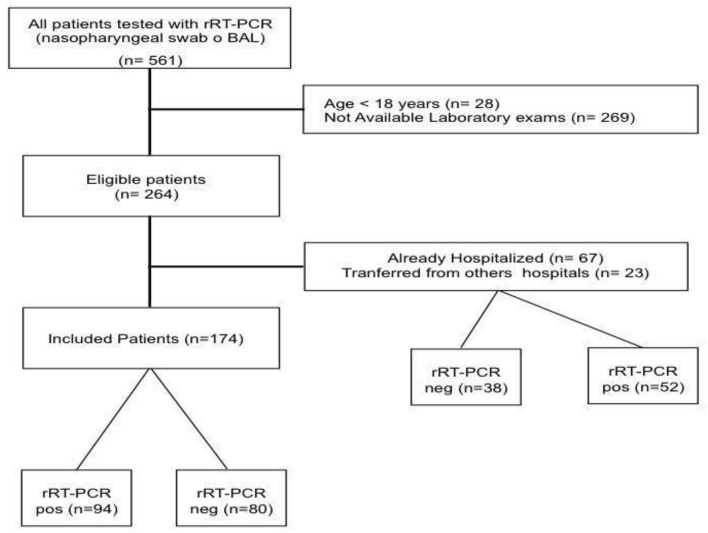
Flow-chart of the study.

Of the 264 patients fulfilling the inclusion criteria, 90 patients were excluded as already hospitalized or transferred from other hospitals (Figure 1); as such, a total of 174 patients were included in the final analysis. Median age of the study population was 58 [43–71] years, with 52% of men and a body mass index (BMI) of 27.5 [23.5–31.6]; the most common reported comorbid disease was arterial hypertension. The median value of the clinical score on admission was 2 [2,3], and the most frequent symptoms were cough (62%), fever (55%) and dyspnea (52%). Among all the tested patients, 94 (54%) had positive rRT-PCR test for SARS-CoV-2 assay on nasopharyngeal swab and/or BAL. The main diagnosis for patients with negative rRT-PCR test are reported in the Table S1. Five patients with an initial negative rRT-PCR test showed positivity to a further test performed at 72 h after admission. Chest CT-scan was performed in 145 (83%) patients; 103/145 (71%) of them showed suggestive findings of COVID-19 related pneumonia. 

### 3.2. Eosinophils and RT-PCR

Eosinophils count on admission was 10 [0–30]/mm^3^ and 134/174 (77%) patients presented eosinopenia. Eosinophils were significantly lower in patients with positive rRT-PCR than others (0 [0–10] vs. 80 [20–180]; *p* < 0.01, Table 1); also, eosinopenia was more frequently observed in patients with positive rRT-PCR than others (89/94 (95%) vs. 45/80, 56%; *p* < 0.01). Patients with anosmia also had more frequently eosinopenia than others, although this was not statistically significant (Table S3).

**Figure 2:**
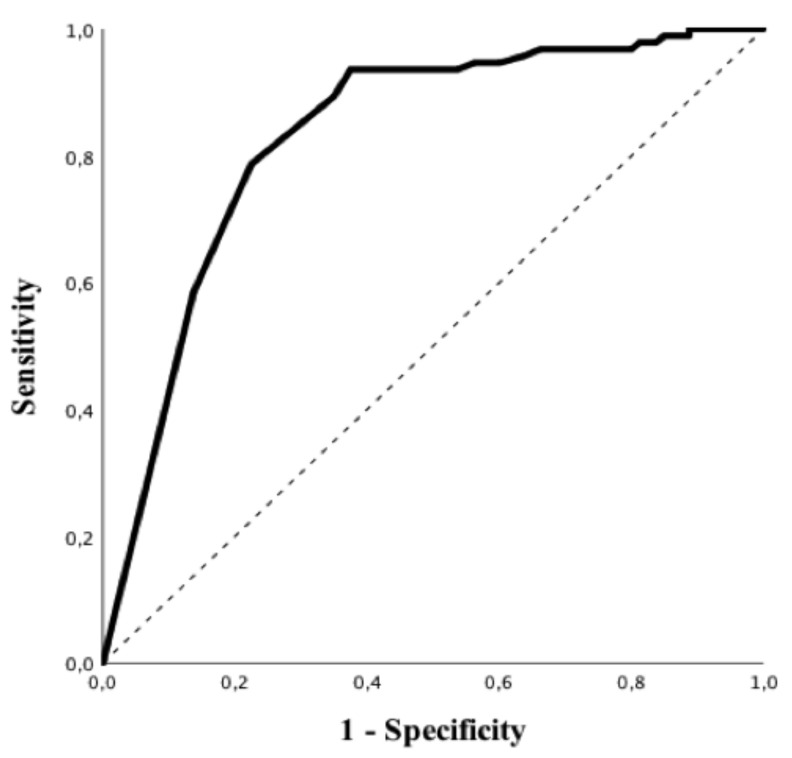
Receiver operating characteristic (ROC) curve of eosinophils counts to predict positive rRT-PCR test for SARS-CoV-2.

Patients with positive rRT-PCR had a higher BMI, higher values of CRP, PT and LDH and lower white blood cells count, albumin levels and lymphocyte to CRP ratio than others (Table 1). In particular, all subtypes of white blood cells were significantly lower in patients with a positive rRT-PCR when compared to those with negative tests. Predictive accuracy of white blood cells subtypes to predict a positive rRT-PCT is shown in Table S2; eosinophils count on admission had the highest predictive value (AUROC 0.84 (95% CIs 0.78–0.90); *p* < 0.01—Figure 2), for positive rRT-PCR among all of them. The Youden’s index identified the threshold of eosinophils of 45/mm^3^ for the best combination of sensitivity (94%) and specificity (63%) to predict positive rRT-PCR. The highest positive predictive value (PPV; i.e., 86%) was observed for an eosinophils count < 5/mm^3^, which was associated with a specificity of 86%, a sensitivity of 59% and a negative predictive value (NPV) of 64%. The highest NPV (i.e., 88%) was observed for an eosinophils count > 100/mm^3^, which was associated with a specificity of 44%, a sensitivity of 95% and a PPV of 66%. In the multivariate analysis, eosinopenia and elevated LDH were independent predictors of positive rRT-PCR (OR 13.42 (95% CIs 4.11–43.77) *p* < 0.01 and OR 1.01 95% [CIs 1.00–1.01] *p* = 0.02, respectively) (Table 3). The eosinophils count on admission was similar between patients with mild/moderate and severe/critical COVID-19 (10 [0–40] vs. 10 [0–40]/mm^3^; *p* = 0.94).

### 3.3. Eosinophils and Chest CT-Scan

Eosinophils count on admission was significantly lower in patients with suggestive chest CT-scan than in others (20 (0–20) vs. 95 (20–170); *p* < 0.01, Table 2); also, eosinopenia was more frequently observed in patients with suggestive chest CT-scan than in others (94/103, 91% vs. 23/42, 55%; *p* < 0.01).

**Figure 3:**
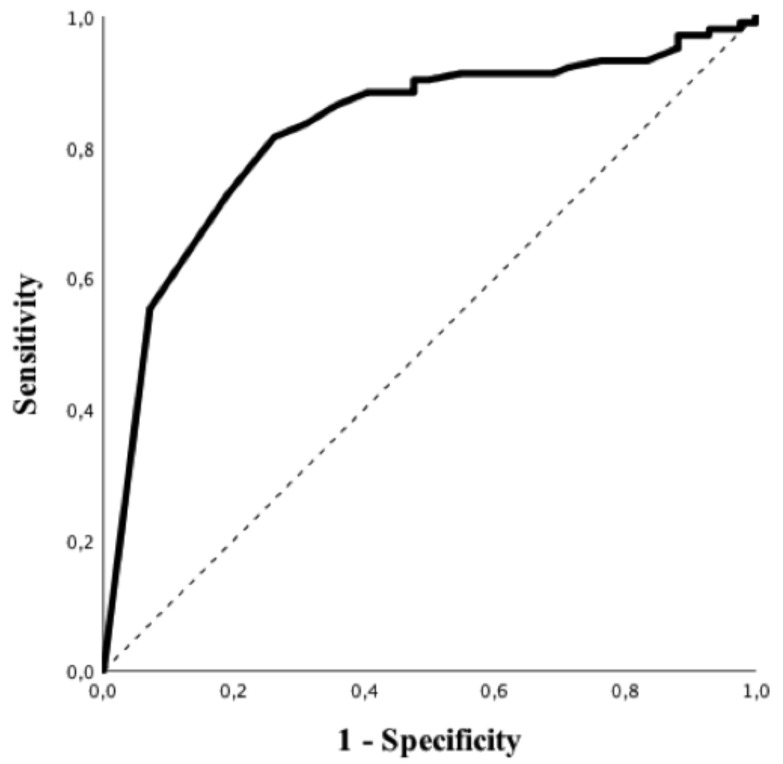
ROC curve of eosinophils counts to predict suggestive CT-scan imaging for COVID-19.

Patients with a suggestive chest CT-scan were more frequently male, had hypertension and a higher BMI and were more frequently admitted to the ICU. In addition, suggestive chest CT-scan was more frequently associated with a positive rRT-PCR (83/103, 81%) vs 7/42, 17%), *p* < 0.01; OR 16.46 (95% CIs 6.49–41.77), *p* < 0.01) and higher levels of CRP, LDH, PT, fibrinogen and LCR, as well as lower albumin levels. As for RT-PCR, all subtypes of white blood cells were significantly lower in patients with a suggestive chest CT-scan when compared to others. Predictive accuracy of white blood cells subtypes to predict a suggestive chest CT-scan is shown in Table S4; eosinophils count on admission had the highest predictive value (AUROC 0.84 (95% CIs 0.77–0.91); *p* < 0.01, Figure 3) to predict a suggestive chest CT-scan among all of them. The Youden’s index identified the threshold of eosinophils of 25/mm^3^ for the best combination of sensitivity (82%) and specificity (74%) to predict a suggestive chest CT-scan. The highest positive predictive value (PPV; i.e., 95%) was observed for an eosinophils count <5/mm^3^, which was associated with a specificity of 93%, a sensitivity of 55% and a NPV of 46%. The highest NPV (i.e., 68%) was observed for an eosinophils count >100/mm^3^, which was associated with a specificity of 45%, a sensitivity of 91% and a PPV of 80%. In the multivariate analysis, high BMI (OR 1.09 [95% CIs 1.01–1.19] *p* = 0.04), arterial hypertension (OR 3.30 [95% CIs 1.29–8.46] *p* = 0.01) and eosinopenia (OR 8.12 [95% CIs 2.61–25.23] *p* < 0.01) were independent predictors of suggestive chest CT-scan for COVID-19 (Table 3). 

## 4. Discussion

In this study, eosinopenia on admission was a common finding in patients with a positive rRT-PCR as well as those with a suggestive chest CT-scan for COVID-19. Eosinophils count had a high predictive accuracy value and was independently associated with both positive rRT-PCR and suggestive chest CT-scan. As such, the combination of eosinophils count on admission and other clinically relevant variables may help physicians to identify patients at risk of COVID-19 if laboratory or imaging resources are lacking.

Eosinophils are circulating and tissue-resident leukocytes, which show proinflammatory, immunoregulatory and antiviral effects [15]. Eosinopenia has been reported in different medical conditions such as bacterial infections, acute inflammation, stress or corticosteroid therapy [16,17,18]. In the setting of COVID-19, eosinopenia may be related to the migration of these cells into the peripheral tissues or to a decreased production of eosinophils into the bone marrow, due to the inflammatory state [16,17,18,19]. Lymphopenia could be another potential explanation for eosinopenia. A large number of patients with COVID-19 present a decreased lymphocytes count, and Th2-subtype lymphocytes are responsible for the production of IL-5, which stimulates the activation and the production of eosinophils [20,21]. In addition, as the eosinophils count can be significantly influenced by the endogenous secretion of glucocorticoids [21], one can argue that increased cortisol levels observed during COVID-19 may result in temporary eosinopenia. Finally, activation of the Fas pathways can induce eosinophil apoptosis (9), which was enhanced by the presence of the Th-1 cytokines, such as IFN-γ and/or TNF-α [22]. We did not specifically assess the causes of eosinopenia and further studies are necessary to investigate these hypotheses in COVID-19 patients.

Recently, Zhang et al. reported that half of the 140 COVID-19 patients admitted to the hospital had eosinopenia (i.e., < 20/mm^3^) on admission^13^; similar results were also reported in another study [23]. In one study (n = 55) including confirmed COVID-19 patients, eosinophil count was associated with poor prognosis [24]. Du et al. reported that in 85 deceased patients with severe COVID-19, 81% of them had absolute eosinophil counts below 20/mm^3^ [25]. In another study, eosinopenia on admission was frequent but eosinophil levels progressively improved over time in all patients prior to discharge, suggesting that resolution of eosinopenia may be an indicator of improving clinical status [26]. A lower eosinophil count on admission was also associated with a significantly longer SARS-CoV-2 positivity on repeated swabs [27]. In another study, Tanni et al. compared two cohort of patients with diagnosis of COVID-19 or Influenza infection and showed that eosinopenia on admission was a good predictor of COVID-19 [28]. Another study also suggested that eosinopenia has an important role to distinguish patients with SARS-CoV-2 infection from other causes of pneumonia [29].

Besides the association of eosinopenia with the presence of COVID-19 or the severity of the disease, one relevant question for the management of COVID-19 is the triage of patients, which has been particularly challenging because of the large number of suspected patients admitted to the hospital and the long waiting time for rRT-PCR results and chest CT-scan availability. Therefore, it is necessary to develop more effective and accessible methods to optimize the triage process of these COVID-19 patients. In one study, the AUROC of eosinophils on admission to predict positive rRT-PCR was 0.77 [95%CI (0.641–0.886); *p*< 0.001], which was lower than the neutrophils-to-lymphocytes ratio and the monocyte-to-lymphocytes ratio [30]; also, the lowest value of eosinophils was observed in severe COVID-19 patients requiring ICU admission. Nevertheless, the authors did not specifically select only patients with available laboratory tests on admission (i.e., also included transfer from another hospital or intrahospital acquisition), which might have influenced final results. In a recent study (n = 989) conducted in Wuhan, eosinopenia (i.e., defined as < 20/mm^3^) was observed in 75% of COVID-19 patients and more frequently (i.e., 31%) than in control patients [31]; eosinopenia had a AUROC of 0.72 to predict positive rRT-PCR. In addition, the combination of eosinopenia and elevated CRP levels increased the predictive accuracy to 0.73. Despite this study not being entirely comparable to our cohort as hospital admission and triage strategy between China and Belgium could have substantially differed, these studies suggest that early eosinophils count assessment, together with other available clinical data, might be useful to identify patients with a possible diagnosis of COVID-19. Eosinophils count is available in routine blood tests worldwide and could facilitate a rapid triage for priority of radio-diagnostic and/or rRT-PCR tests. 

Very few diagnostic scores are available to rapidly identify patients with a high risk of positive rRT-PCR testing during COVID-19 pandemic. In one study, history of close contact, fever, neutrophil-to-lymphocyte ratio, high temperature and gender were included into a “COVID-19 early warning score”, which had a AUROC to diagnose a positive rRT-PCR of 0.96 [32]; however, chest CT-scan was also included in the final model. In a systematic review evaluating diagnostic models to detect COVID-19 infection in patients with symptoms, ten prognostic models including clinics, routine laboratory tests and other clinical information on admission were identified, with variable predictive accuracy and calibration [33]; their applicability in clinical practice remains therefore to be further evaluated. In addition, no reliable or useable clinical score currently exists to predict outcomes or inform decisions regarding hospital admission for COVID-19 patients.

This study has several limitations. First, this is a retrospective, single-centre study; as such, the results may suffer from selection biases. Moreover, we did not calculate the sample size to test the diagnostic accuracy of eosinopenia to predict a positive rRT-PCR test as, at the moment the study was initiated, the data on eosinopenia in COVID-19 were very limited and the prevalence of the positive rRT-PCR test in patients presenting in our institution was unpredictable. In particular, for a prevalence estimated around 50% of positive rRT-PCR testing, a minimum sample size of 365 patients would have been required to achieved a power of 80% in order to detect a change in the sensitivity from 0.80 to 0.90 and a change in the specificity from 0.6 to 0.7, with a significance level of 0.05. Future and multicentric studies are warranted to confirm our results using larger cohorts of patients. Second, some relevant laboratory variables might have been missed. Third, the sample size was not calculated and external validation not provided. Fourth, rRT-PCR could yield false negative results, mainly due to an insufficient viral load below the detection limit or inadequately collected nasopharyngeal swabs. Fifth, the clinical score was used locally and has not been validated in larger cohorts. Finally, the “pre-test” prevalence of COVID-19 in the analysed cohort could be a significant confounder to extrapolate the validity of our findings in clinical practice. Giving the high prevalence of COVID-19 among patients (i.e., the study was carried out during the epidemic phase), it is difficult to conclude that eosinopenia is always related to a positive RT-PCR test for SARS-Cov-2. Indeed, these data should be re-evaluated during a phase of lower COVID-19 prevalence, where other inflammatory diseases could also be associated to initial eosinopenia and contribute as false positive. Moreover, we evaluated only hospitalized patients and the conclusions of our study cannot be applied to mild COVID-19 forms. 

## 5. Conclusions

In this study, eosinopenia was frequently observed in COVID-19 patients and could be helpful to identify patients with positive rRT-PCR testing or suspected chest CT-scan findings. This easily accessible parameter should be considered in the triage process to speed up the diagnosis and treatment of COVID-19 patients.

## Figures and Tables

**Table 1:** Characteristics of the study population, according to rRT-PCR results on admission.

	Overall (n = 174)	rRT-PCR Positive (n = 94)	rRT-PCR Negative (n = 80)	*p* Value
Age, years	58 [43–71]	58 [46–67]	65 [39–74]	0.99
Male, n (%)	90 (52)	54 (57)	36 (45)	0.13
BMI, kg/m^2^	27.5 [23.5–31.6]	28.9 [25–31.8]	25.6 [22.9–31]	0.02
Hypertension, n (%)	77 (45)	46 (49)	31 (40)	0.28
Diabetes, n (%)	48 (28)	30 (32)	18 (23)	0.23
Heart Disease, n (%)	39 (22)	19 (20)	20 (26)	0.47
Previous Neurologic Disease, n (%)	28 (16)	14 (15)	14 (18)	0.68
Chronic Kidney Disease, n (%)	32 (18)	19 (20)	13 (17)	0.69
Asthma/COPD, n (%)	32 (18)	13 (14)	19 (24)	0.11
Autoimmune Disease, n (%)	18 (10)	10 (11)	8 (10)	1
Allergies, n (%)	31 (18)	16 (17)	15 (19)	0.84
Cancer, n (%)	27 (16)	10 (11)	17 (22)	0.06
Psychiatric Disease, n (%)	17 (10)	7 (7)	10 (13)	0.3
Liver Cirrhosis, n (%)	6 (3)	3 (3)	3 (4)	1
Alcohol, n (%)	13 (8)	8 (9)	5 (6)	0.77
Active Smoking, n (%)	30 (17)	13 (14)	17 (22)	0.23
Immunosuppressive Therapy, n (%)	22 (13)	13 (14)	9 (12)	0.82
NSAIDs, n (%)	21 (12)	8 (9)	13 (17)	0.16
ARB-ACE, n (%)	39 (22)	26 (28)	13 (17)	0.1
Corticosteroids, n (%)	27 (15)	14 (15)	13 (17)	0.83
Fever on admission (%)	96 (60)	64 (70)	32 (48)	<0.01
Cough on admission (%)	107 (68)	65 (71)	42 (63)	0.30
Dyspnea on admission (%)	90 (57)	59 (66)	31 (46)	0.01
Anosmia on admission (%)	11 (6)	10 (11)	1 (1)	0.03
Bacterial co-infection on admission	25 (15)	11 (12)	14 (18)	0.39
Viral co-infection on admission	6 (4)	1 (1)	5 (6)	0.07
Time from symptoms to Hospital, days	6 [2–7]	7 [4–8]	3 [1–7]	<0.01
Time from admission to test, days	0 [0–1]	1 [0–1]	0 [0–1]	0.08
ICU admission, n (%)	18 (10)	13 (14)	5 (6)	0.13
Suggestive chest CT-scan, n (%)	103 (59)	79 (92)	24 (41)	<0.01
Clinical score on admission	2 [2–3]	2 [2–3]	2 [1–2]	<0.01
PT on admission, %	88 [78–96]	93 [85–98]	85 [74–96]	0.01
Fibrinogen on admission, mg/dL	475 [361–593]	505 [400–634]	438 [351–522]	0.23
WBC on admission, n/mm^3^	10430 [8230–11340]	5750 [4470–7470]	9820 [6550–12660]	<0.01
RBC on admission, n*10⁶/mm^3^	4.68 [4.11–5.11]	4.85 [4.19–5.19]	4.66 [3.97–4.94]	0.19
Haemoglobin on admission, g/dL	13.6 [11.6–14.6]	13.9 [11.8–14.7]	13.2 [11.2–14.2]	0.14
RDW on admission, %	13.45 [12.7–14.8]	13.2 [12.6–14.3]	13.9 [13–16.2]	<0.01
Platelets on admission, n*10^3^/mm^3^	209 [163–273]	188 [152–237]	251 [175–311]	<0.01
Neutrophils on admission, n/mm^3^	4910 [340–7840]	4240 [3160–5760]	6690 [4590–10030]	<0.01
Lymphocytes on admission, n/mm^3^	1160 [730–1740]	920 [690–1380]	1440 [840–2190]	<0.01
Monocytes on admission, n/mm^3^	550 [330–780]	430 [30–580]	680 [480–900]	<0.01
Eosinophils on admission, n/mm^3^	10 [0–30]	0 [0–10]	80 [20–180]	<0.01
Basophils on admission, n/mm^3^	10 [10–30]	10 [0–10]	30 [20–50]	<0.01
NLR on admission	4.08 [2.59–7.31]	4.82 [3.01–7.13]	3.79 [2.5–8.43]	0.81
LCR on admission	0.03 [0.01–0.14]	0.01 [0.01–0.05]	0.07 [0.02–0.42]	<0.01
C-Reactive Protein on admission, mg/L	53 [13–110]	72 [22–120]	19 [5–71]	<0.01
Procalcitonine on admission, mcg/L	0.11 [0.05–0.29]	0.12 [0.05–0.28]	0.09 [0.05–0.32]	0.78
Urea on t admission, mg/dL	33.2 [21.9–48.3]	34.4 [22.4–50.6]	32.6 [21.5–47.4]	0.76
Creatinine on admission, mg/dL	0.95 [0.77–1.34]	1 [0.8–1.33]	0.9 [0.74–1.36]	0.19
Bilirubin on admission, mg/dL	0.48 [0.33–0.61]	0.47 [0.33–0.6]	0.51 [0.32–0.65]	0.3
SGPT on admission, UI/L	23 [14–40]	29 [16–43]	19 [11–31]	<0.01
SGOT on admission, UI/L	28 [20–50]	40 [26–55]	22 [17–41]	<0.01
LDH on admission, UI/L	272 [190–394]	282 [214–417]	241 [175–336]	0.02
Troponin on admission, ng/L	12 [6–28]	12 [6–24]	13 [7–33]	0.39
Albumin on admission, g/L	40 [37–42]	39 [35–42]	42 [38–43]	<0.01

BMI = body mass index; COPD = chronic obstructive pulmonary disease; rRT-PCR = reverse real time polymerase chain reaction, NSAIDs = non-steroidal anti-inflammatory drugs; ICU = intensive care unit; CT = computerized tomography; PT = prothrombin time; WBC = white blood cell; RBC = red blood cell; RDW = red cell distribution width; NLR = neutrophil to lymphocyte ratio; LCR = lymphocyte to C-reactive protein ratio; SGOT = serum glutamic oxaloacetic transaminase; SGPT = serum glutamate-pyruvate transaminase; LDH = lactate dehydrogenase. Data are expressed as counts (percentage) or median [interquartile range].

**Table 2:** Characteristics of study population, according chest CT-scan results on admission.

	CT Scan Suggestive (n = 103)	CT Scan Not Suggestive (n = 42)	*p* Value
Age, years	60 [49–71]	65 [43–74]	0.98
Male, n (%)	61 (59)	15 (36)	0.01
BMI, kg/m^2^	29.1 [25.0–32.4]	24.4 [23.1–28.8]	<0.01
Hypertension, n (%)	56 (55)	13 (31)	0.01
Diabetes, n (%)	34 (33)	13 (31)	0.85
Heart Disease, n (%)	22 (22)	14 (33)	0.15
Previous Neurologic Disease, n (%)	16 (16)	8 (19)	0.63
Chronic Kidney Disease, n (%)	20 (20)	10 (24)	0.65
Asthma/COPD, n (%)	16 (16)	12 (29)	0.10
Autoimmune Disease, n (%)	10 (10)	5 (12)	0.77
Allergies, n (%)	17 (17)	9 (21)	0.49
Cancer, n (%)	12 (12)	13 (31)	<0.01
Psychiatric Disease, n (%)	11 (11)	5 (12)	0.99
Liver Cirrhosis, n (%)	3 (3)	3 (7)	0.36
Alcohol, n (%)	9 (9)	4 (10)	0.99
Active Smoking, n (%)	15 (15)	11 (26)	0.15
Immunosuppressive Therapy, n (%)	14 (14)	6 (14)	0.99
NSAIDs, n (%)	13 (13)	6 (14)	0.79
ARB-ACE, n (%)	29 (28)	8 (19)	0.30
Corticosteroids, n (%)	15 (15)	10 (24)	0.23
Fever on admission (%)	67 (67)	16 (47)	0.04
Cough on admission (%)	71 (72)	19 (56)	0.10
Dyspnea on admission (%)	63 (64)	20 (59)	0.68
Anosmia on admission (%)	10 (10)	0 (0)	0.06
Bacterial co-infection on admission	11 (11)	12 (29)	0.01
Viral co-infection on admission	3 (3)	1 (2)	1.00
ICU admission, n (%)	15 (15)	1 (3)	0.04
rRT-PCR positive Test, n (%)	83 (81)	7 (17)	<0.01
Clinical score on admission	2 [2–3]	2 [1–2]	<0.01
PT on admission, %	91 [83–98]	85 [73–95]	0.03
Fibrinogen on admission, mg/dL	505 [417–634]	422 [348–483]	0.01
WBC on admission, n/mm^3^	5900 [4730–8710]	9080 [6310–12520]	<0.01
RBC on admission, n*10⁶/mm^3^	4.80 [4.29–5.15]	4.46 [3.96–4.80]	0.01
Haemoglobin on admission, g/dL	13.9 [11.9–14.7]	12.8 [10.9–14.0]	0.04
RDW on admission, %	13.3 [12.7–14.7]	14.1 [13.2–16.5]	0.03
Platelets on admission, n*10^3^/mm^3^	190 [151–245]	243 [173–309]	0.02
Neutrophils on admission, n/mm^3^	4.510 [3220–6610]	6160 [4670–10000]	<0.01
Lymphocytes on admission, n/mm^3^	1.040 [730–1430]	1150 [660–2210]	0.12
Monocytes on admission, n/mm^3^	430 [300–620]	650 [500–870]	<0.01
Eosinophils on admission, n/mm^3^	0 [0–20]	95 [20–170]	<0.01
Basophils on admission, n/mm^3^	10 [0–20]	30 [20–50]	<0.01
NLR on admission	4.17 [2.87–7.50]	4.39 [2.49–9.63]	0.92
LCR on admission	0.02 [0.01–0.05]	0.10 [0.01–0.44]	<0.01
C-Reactive Protein on admission, mg/L	70 [17–115]	13 [4–56]	<0.01
Procalcitonine on admission, mcg/L	0.12 [0.07–0.29]	0.11 [0.05-–0.38]	0.51
Urea on t admission, mg/dL	38 [24–52]	32 [22–82]	0.71
Creatinine on admission, mg/dL	1.00 [0.84–1.38]	0.96 [0.73–1.59]	0.97
Bilirubin on admission, mg/dL	0.49 [0.33–0.61]	0.44 [0.32–0.71]	0.78
SGPT on admission, UI/L	25 [15–41]	21 [13–32]	0.15
SGOT on admission, UI/L	39 [24–52]	25 [17–45]	0.01
LDH on admission, UI/L	309 [246–415]	219 [170–289]	<0.01
Troponin on admission, ng/L	13 [9–29]	15 [6–35]	0.74
Albumin on admission, g/L	39 [36–42]	40 [39–44]	0.04

BMI = body mass index; COPD = chronic obstructive pulmonary disease; rRT-PCR = reverse real time polymerase chain reaction, NSAIDs = non-steroidal anti-inflammatory drugs; ICU = intensive care unit; CT = computerized tomography; PT = prothrombin time; WBC = white blood cell; RBC = red blood cell; RDW = red cell distribution width; NLR = neutrophil to lymphocyte ratio; LCR = lymphocyte to C-reactive protein ratio; SGOT = serum glutamic oxaloacetic transaminase; SGPT = serum glutamate-pyruvate transaminase; LDH = lactate dehydrogenase. Data are expressed as counts (percentage) or median [interquartile range].

**Table 3:** Multivariable logistic regression to predict rRT-PCR positive or suggestive chest CT-scan.

	OR [95% CIs]	*p* Value
rRT-PCR positive for SARS-CoV-2		
Eosinopenia	13.42 [4.11–43.77]	<0.01
LDH, IU/L	1.01 [1.00–1.01]	0.02
CT Scan suggestive for COVID-19		
BMI, Kg/m^2^	1.09 [1.01–1.19]	0.04
Arterial Hypertension	3.30 [1.29–8.46]	0.01
Eosinopenia	8.12 [2.61–25.23]	<0.01

BMI = body mass index; rRT-PCR = reverse real time polymerase chain reaction, LDH = lactate dehydrogenase; OR = odds ratio; CI = confidence intervals. Data indicate adjusted odds ratios [95% confidence intervals].

## Supplementary Materials

The following are available online at https://www.mdpi.com/2076-2607/8/12/1929/s1.
