# Supplementary material for: Eosinopenia in COVID-19 Patients: A Retrospective Analysis
Source: Microorganisms. 2020 Dec 4;8(12):1929. doi: 10.3390/microorganisms8121929 (PMC7762070; doi:10.3390/microorganisms8121929)
Supplement: Supplementary file 1 [file microorganisms-08-01929-s001.pdf]

Table S1. Diagnosis of patients with rRT-PCR test negative.

Diagnosis	n = 80
Others pulmonary infections, n (%)	12 (15)
COPD/Asthma, n (%)	13 (16)
Pulmonary Embolism, n (%)	2 (2)
Acute Cardiac Disease, n (%)	11 (14)
Acute Neurologic Disease, n (%)	3 (4)
Cancer, n (%)	8 (10)
Others not pulmonary infections, n (%)	7 (9)
Others minor diagnosis (i.e. Anxiety, Psychosis) , n (%)	24 (30)

COPD = Chronic obstructive pulmonary disease

Table S2. Area under the ROC curve (AUROC) for subtypes of white blood cells on admission to predict positive rRT-PCR.

	AUROC	C.I. 95%	*p*-value
White Blood Cells	0.77	0.70–0.84	< 0.01
Neutrophils	0.70	0.62–0.78	< 0.01
Lymphocytes	0.67	0.60–0.76	< 0.01
Monocytes	0.73	0.65–0.80	< 0.01
Eosinophils	0.84	0.78–0.90	< 0.01
Basophils	0.81	0.74–0.88	< 0.01
Neutrophils to Lymphocytes ratio	0.51	0.43–0.60	0.76
Lymphocytes to CRP ratio	0.72	0.64–0.79	< 0.01

CRP = C-Reactive Protein; 95% CI = Confidence Interval 95%.

Table S3. Number of patients with different levels of eosinopenia, according to presence of anosmia.

	Anosmia (n = 11)	Not Anosmia (n = 163)	*p-value*
Eosinophils < 100/mm^3, n (%)	11 (100)	123 (76)	0.07
Eosinophils < 45/mm^3, n (%)	11 (100)	107 (66)	0.01
Eosinophils < 5/mm^3, n (%)	7 (64)	59 (36)	0.11

Table S4. Area under the ROC curve (AUROC) for subtypes of white blood cells on admission to predict suspected chest CT-scan findings.

	AUROC	C.I. 95%	*p value*
White Blood Cells	0.70	0.60–0.80	< 0.01
Neutrophils	0.65	0.55–0.76	< 0.01
Lymphocytes	0.60	0.49–0.71	0.07
Monocytes	0.71	0.6 –0.80	< 0.01
Eosinophils	0.84	0.77–0.91	< 0.01
Basophils	0.84	0.77–0.91	< 0.01
Neutrophils to Lymphocytes ratio	0.51	0.40–0.62	0.91
Lymphocytes to CRP ratio	0.71	0.61–0.81	< 0.01

CRP = C-Reactive Protein; 95% CI = Confidence Interval 95%.
